# Cancroin Identical with Neurin

**Published:** 1913-02

**Authors:** 


CANCROIN IDENTICAL WITH NEURIN.—Adamiewicz (quoted, ibid.) having apparently found injections of cancer extract beneficial in the treatment of cancer, and believing the active principle to be identical with neurin, advises the following formula:

25% solution of neurin 10 parts.
Saturated sol. citric acid 1.82 parts.
Saturated sol. phenol 1.25 parts.
Water to 30. parts.
This is further diluted with water 50%, and 1 c. c. is injected.

